# Digitization of Broccoli Freshness Integrating External Color and Mass Loss

**DOI:** 10.3390/foods9091305

**Published:** 2020-09-16

**Authors:** Yoshio Makino, Genki Amino

**Affiliations:** Graduate School of Agricultural and Life Sciences, The University of Tokyo, 1-1-1, Yayoi, Bunkyo-ku, Tokyo 113-8657, Japan; north.big.place@gmail.com

**Keywords:** *Brassica oleracea* var. *italica*, computer vision, machine learning, evaluation, shelf life, statistical analysis, vegetable, image analysis

## Abstract

Yellowing of green vegetables due to chlorophyll decomposition is a phenomenon indicating serious deterioration of freshness, and it is evaluated by measuring color space values. In contrast, mass reduction due to water loss is a deterioration of freshness observed in all horticultural crops. Therefore, in this study, we propose a novel freshness evaluation index for green vegetables that combines the degree of greenness and mass loss. The green color retention rate was measured using a computer vision system, and the mass retention rate was measured by weighing. Linear discriminant analysis (LDA) was performed using both variables (greenness and mass) as covariates to obtain a single freshness evaluation value (first canonical variable). The correct classification of storage period length by LDA was 96%. Green color retention alone allowed for classification of storage durations between 0 day and 10 days, whereas LDA could classify storage durations between 0 day and 12 days. The novel freshness evaluation index proposed by this research, which integrates greenness and mass, has been shown to be more accurate than the conventional evaluation index that uses only greenness.

## 1. Introduction

The freshness of horticultural crops continuously declines after harvest, and the commodities eventually lose their commercial viability, resulting in food loss [1]. It is quite difficult to store fresh vegetables and maintain their freshness after they have been harvested, and the loss/waste rate of vegetables during the distribution process is 18% in developed countries and 46% in developing countries [2]. Loss of horticultural crops means that all resources such as seeds, fertilizers, pesticides, and other agricultural materials input for production, energy for operating agricultural machinery, labor required for agricultural work, etc. are wasted. Therefore, reduction of the loss/waste rate is an important issue from the viewpoint of sustainable food production and improvement of food cycle. Various freshness preservation methods are used to reduce the loss of horticultural crops. Li et al. [3] reported the effect of retaining visual acceptability among refrigerated produce, and suppressing mass loss and decay for eight types of items stored at 6 °C. Controlled atmosphere (CA) storage, which suppresses respiration under low O_2_ and high CO_2_ environments, demonstrates a significant freshness-keeping effect when used in combination with refrigeration, and is employed for the long-term storage of apples [4]. A storage unit is harnessed for CA storage. In contrast, modified atmosphere packaging (MAP), which is obtained by achieving the same effect using a plastic pouch, stops the spoilage of tomatoes [5], the softening of persimmons [6], and suppresses the yellowing of soft kale [7] and vegetable soybeans [8]. The inactivation of ethylene gas as an aging hormone is an effective freshness-keeping method, and a high number of freshness effects derived from the ethylene-inactivating regent “1-methylcyclopropene” were reported as introduced in a review by Watkins [9]. Heat treatment has been identified as possessing the effect of sterilizing microorganisms, deactivating enzymes, and inducing heat shock protein to maintain freshness in vegetables [10]. Akbudak et al. [11] reported that heating vegetables at 54 °C for 5 min was effective for preserving the freshness of tomatoes. The freshness of fish is objectively evaluated by a K-value calculated from changes in nucleic acid composition after death [12]. In contrast, the freshness of horticultural crops does not have defined universal evaluation criteria, and post-harvest condition is determined using measured values of evaluation criteria that are closely related to freshness [13]. For example, horticultural crops lose water through transpiration after harvest, resulting in the deterioration of external quality factors such as wilting and loss of water [14]. In addition, overall quality [13], fruit hardness [15], cell membrane deterioration [16], L-ascorbate content [7] and spoilage [17] are reported as freshness evaluation indices. Discoloration is an evaluation index indicating deterioration of appearance due to loss of freshness. Browning of the surface of white vegetables such as cauliflower (*Brassica oleracea* var. *botrytis*) and the cross-sections of cut vegetables is mainly due to an oxidation reaction catalyzed by polyphenol oxidase (EC. 1.10.3.1) and phenylalanine ammonia lyase (EC. 4.3.1.24) [18]. A more serious external color change as freshness decreases is the yellowing of green vegetables caused by the decomposition of the green pigment chlorophyll and the yellow color of the remaining carotenoids [19]. The appearance of deterioration due to yellowing has been studied in edamame (*Glycine max* (L.) Merr.) [8] and soft kale (*Brassica oleracea* L. var. *acephala* DC.) [7]. In this study, a freshness evaluation index for broccoli (*Brassica oleracea* var. *italica*), which has a high respiration rate and marked yellowing after harvest [20,21], was proposed. Robinson et al. [21] reported that commodities that respired faster had a shorter freshness retention period. Cellular respiration is a metabolic reaction that transmits the extracted H^+^ and e^−^ while depleting nutrients such as carbohydrates, lipids and organic acids, and produces the high-energy compound “Adenosine 5’-triphosphate” necessary for metabolic activities [22]. In order to eliminate H^+^ generated during the process, it is oxidized by O_2_ molecules taken up into cells to generate water [23]. Plant cells such as vegetables have two types of terminal oxidases (cytochrome *c* oxidase: COX; alternative oxidase: AOX), and nutrients are depleted as a result of oxidation, resulting in deterioration of freshness. Makino et al. [24] reported that although the amount of AOX induction increased with the decrease in freshness, the amount of COX induction was stable. Additionally, Wang et al. [25] reported that O_2_ consumption by AOX associated with respiration promoted chlorophyll decomposition, as broccoli yellowing proceeded with increasing AOX induction. The yellowing of broccoli is due to the decomposition of the green pigment chlorophyll with decreased freshness, making the yellow color of carotenoids more conspicuous. One type of peroxidase (EC. 1.11.1.x) has been reported to be responsible for chlorophyll decomposition [19].

Studies in which the appearance color of a horticultural crop was evaluated using an index calculated from CIE 1976 L*a*b* [26] have been reported. Yokota et al. [27] evaluated the change from green to red in a*/b* during the ripening process of tomato. Makino et al. [28] classified mango grades based on hue angle (tan^−1^ (b*/a*)). Li et al. [29] assessed postharvest yellowing of green soybeans using hue angle. In contrast, the yellowing of broccoli is often evaluated by the value −a*/b* [30]. Wang et al. [25] evaluated −a*/b* with a colorimeter and objectively evaluated controlled atmosphere storage and modified atmosphere packaging on the green color retention of broccoli. Makino et al. [31] used a computer vision system (CVS) to visualize the temporal changes in the two-dimensional spatial distribution of green color retention associated with decreased freshness of broccoli flower buds. In addition to yellowing, a significant decrease in mass was also observed [32]. Similar to yellowing, mass loss was promoted as the respiration rate increased, and the O_2_ consumption rate of broccoli stored under a low O_2_ and high CO_2_ environment was suppressed compared to the sample stored under normoxia, and the mass reduction was also suppressed accordingly [24]. It was confirmed that the induction of AOX was suppressed under the environment of low O_2_ and high CO_2_, and that the suppression of respiration contributed to the preservation of freshness, that is, the suppression of mass loss [24]. From the above findings, it is clear that the decrease in freshness is promoted by respiration (O_2_ consumption) and is observed as yellowing and mass loss of green vegetables such as broccoli. Therefore, in this study, we devised a novel freshness evaluation index which integrates yellowing and mass loss and verified its effectiveness.

## 2. Materials and Methods

### 2.1. Materials

Nine heads of broccoli (cultivar Pixel) were harvested from a farm in Saitama Prefecture. They were transported to the laboratory under a low-temperature environment (5 °C–10 °C) within one day of harvest. The main stems were cut, and the sample heights were adjusted to 12 cm. After storing at 10 °C, samples were used for the following experiments one day after harvest.

### 2.2. Storage Method

Samples were stored in a thermo-hygrostat (FLI-301NH, EYELA Co., Ltd., Tokyo, Japan) for 14 days at 10 °C and 80% relative humidity (R.H.). Freshness was measured every 2 days during the storage period. External color was measured with a CVS [31], and mass was measured with an electronic balance (MS4002S, Mettler Toledo International Inc., Tokyo, Japan). The data were obtained for nine biological samples at eight storage timepoints from 0 day to 14 days (every 2 days), and a total of 72 data points were obtained. These data were divided into a calibration data set (48 data) and a test data set (24 data) and subjected to the machine learning mentioned in Section 2.3.

### 2.3. Digitization of Freshness

The green color retention of each sample was quantified by the method of Makino et al. [31]. Specifically, the images (Windows bitmap format) acquired by the CVS were imported into MATLAB ver. 9.8.0.1323502 (Mathworks Inc., Natick, MA, USA), the backgrounds were removed, and the mean CIE 1976 L*a*b* color space values per image were calculated. In accordance with previous research [25,30], the green color was evaluated as −a*/b*. CVS was composed of a digital camera (FMVU-13S2C-CS, Point Gray Research Inc., Richmond, BC, Canada) with lens (13FM06IR, Tamron Co., Ltd., Saitama, Japan) which was fixed to the central pole of a color viewer (PIAS IS-500, Sugiura Laboratory Inc., Tokyo, Japan). This color viewer was equipped with six 10 W fluorescent lamps with a color temperature of 6500 K (D_65_) as the light source. The angle between the camera lens and lighting source axis was approximately 45°, the same as that used by Mendoza et al. [33].

Mass loss was evaluated using mass retention rate [8], which is an index for evaluating the freshness of vegetables. Water loss is reported to be the major cause of decreased mass retention [14]. Furthermore, it has been reported that plant size decreases with decreasing water content [34]. The above findings suggest that it is possible to estimate the mass retention rate by image analysis. Therefore, in this study, we utilized a method for determining the mass retention rate using images. We propose a mathematical formula that associates mass retention rate with the retention rate of the flower bud area (number of pixels) and associates the mass retention rate from the area retention rate. The digitization of freshness is calculated using the following formula:

*v_r_* = *v_t_*/*v*_0_, (1)

where, *v* is green color (−a*/b*), mass (g) or area (pixel), subscript *r* is retention rate, *t* is storage period, and 0 is the initial value.

Partial least square discriminant analysis (PLS-DA) [35] was performed using the combination of green color retention rate and either mass retention rate or area retention rate from the calibration data set. Linear discriminant analysis (LDA) [36] was also performed using the combination of green color retention rate and either mass retention rate or area retention rate from the calibration data set. A single index (PLS-DA: latent variable, LV; LDA: canonical variable, CV) was calculated by integrating the green color retention rate and mass (or area) retention rate during the storage period of 0 day to 14 days. A freshness digitization model was created to classify images by correct storage period length.

*F_i_* = *f*(*G*, *M* or *A*), (2)

where *A* is area retention rate, *f* is a function created by PLS-DA or LDA, *F_i_* is the novel fresh evaluation index, *G* is green color retention rate, and *M* is mass retention rate.

The model created using the calibration data set was applied to the test data set, and the storage period lengths were predicted by the proposed mathematical models.

In addition, JMP^®^ Pro ver. 15.0.0 (SAS Institute Inc., Cary, NC, USA) was used for the calculation of analysis of variance (ANOVA), PLS-DA, and LDA.

## 3. Results

### 3.1. Evaluation of Freshness by Conventional Methods

**Figure 1:**
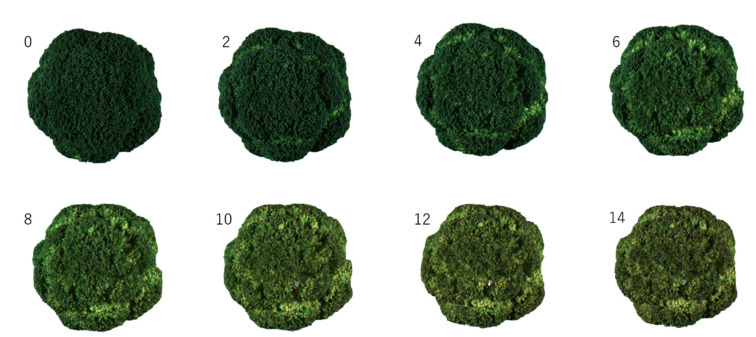
Images of typical broccoli heads stored at 10 °C and 80% relative humidity for 0 day to 14 days. The numbers denote the duration of storage period (day).

Images of a representative sample are shown in Figure 1. The green color of the sample was lost over time, fading became apparent at approximately day 4, and yellowing became noticeable on and after day 8. The area of the flower bud tended to shrink over time. This was thought to be due to water loss [34].

**Figure 2:**
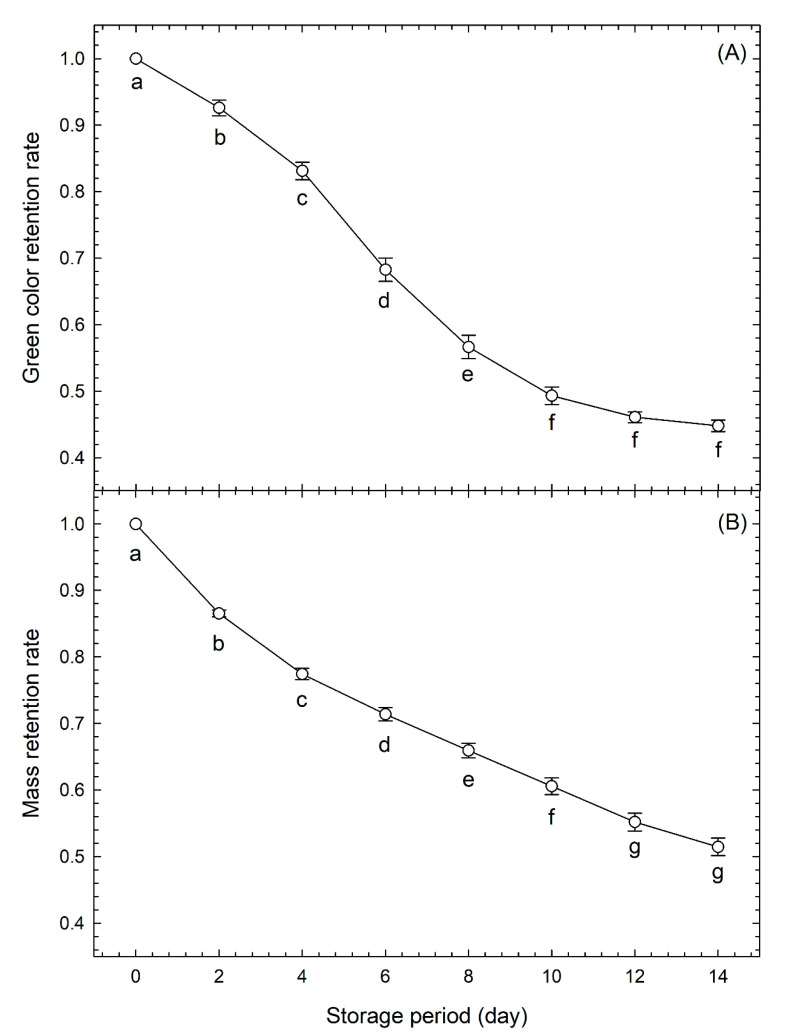
Changes in (**A**) green color (−a*/b*) retention and (**B**) mass retention rates of broccoli heads stored at 10 °C and 80% relative humidity over time. Values are the means ± SE of six observations from six biological samples (calibration data set). Symbols followed by the same letter within the same figure indicate no significant difference (*p* < 0.05, Tukey’s honest difference test).

Figure 2A shows the changes in the green color retention rate over time of the calibration data set. The green color retention rate decreased significantly every 2 days between day 0 and day 10; however, there was no significant difference in the values between day 10 and day 14. We propose that this was because yellowing had progressed considerably up to day 10, and the difference in yellowness was difficult to detect thereafter.

Figure 2B shows the changes in mass retention rates of the calibration data set over time. There was a significant decrease in mass retention every 2 days between day 0 and day 12; however, there was no significant difference between the values from day 12 and day 14. We propose that this was because the mass had reduced considerably up to day 12, and the difference in mass was difficult to detect thereafter.

**Figure 3:**
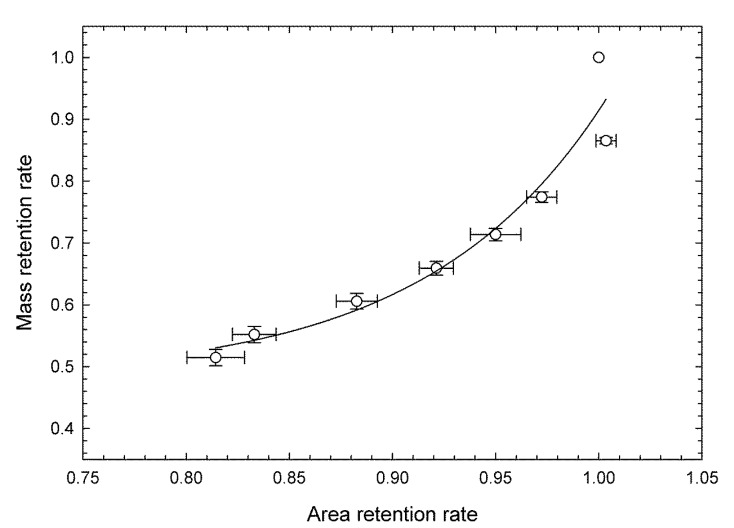
Relationship between mass retention and area retention rates of broccoli heads stored for 14 days at 10 °C and 80% relative humidity. Values are the means ± SE of six observations from six biological samples (calibration data set). Regression curve: *y* = 0.479 + 4.27 × 10^−6^·exp(11.5·*x*), correlative coefficient = 0.965, standard error of calibration = 0.0508.

Figure 3 shows the relationship between mass retention and area retention rates (both calibration data set). It was approximated by an exponential function, and the correlation coefficient was high, however the relationship was non-linear. That is, the amount of change in area per amount of change in mass retention tended to be small at the beginning of storage and increased as the length of the storage period increased. In contrast, since the correlation coefficient was high, it was suggested that the mass retention rate could be estimated from the area retention rate, and thus the mass retention rate could be predicted by image capturing.

Table 1 shows the results of ANOVA of the effect of storage period duration on the retention rates for green color, mass, and area. All variables were significantly affected by the length of the storage period at a 99.9% level and were clearly shown to decrease over time, supporting the results shown in Figure 2 and Figure 3.

### 3.2. Evaluation of Freshness by a Novel Evaluation Index Integrating Green Color, Mass, and Area Retention Rate

**Figure 4:**
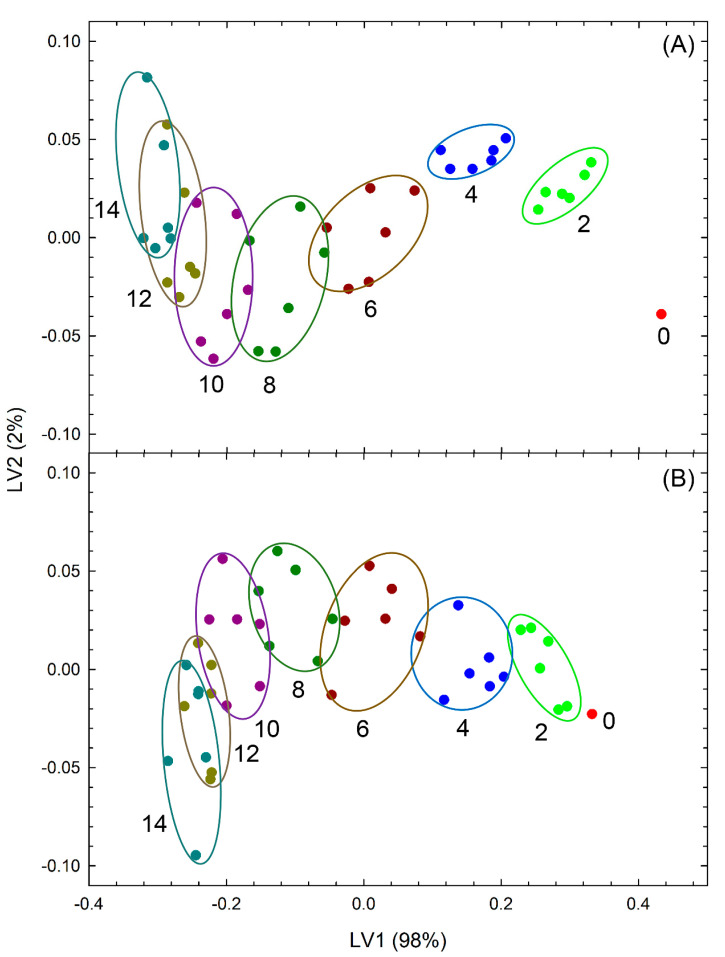
Partial least square discriminant analysis score plots of the freshness of broccoli heads (calibration data set) stored at 10 °C and 80% relative humidity. (**A**) input variables: green color and mass retention rate, (**B**) input variables: green color and area retention rate, LV denotes the latent variable, numbers in the figure denote the storage period length in days; the difference of colors of the plots and circles in this figure denotes the difference of storage period duration.

Figure 4 shows the results from PLS-DA (score plot) using the calibration data set with green color retention rate and mass retention rate or area retention rate as input variables classified by the length of the storage periods. The input variables were green color retention and mass retention rate (Figure 4A) or green color retention and area retention rate (Figure 4B). In these cases, the score values corresponded to the integrated freshness evaluation index. The score values tended to decrease with the length of the storage periods; therefore, LV1 was considered to be a variable closely related to storage period length, and the contribution rate was 98%.

**Figure 5:**
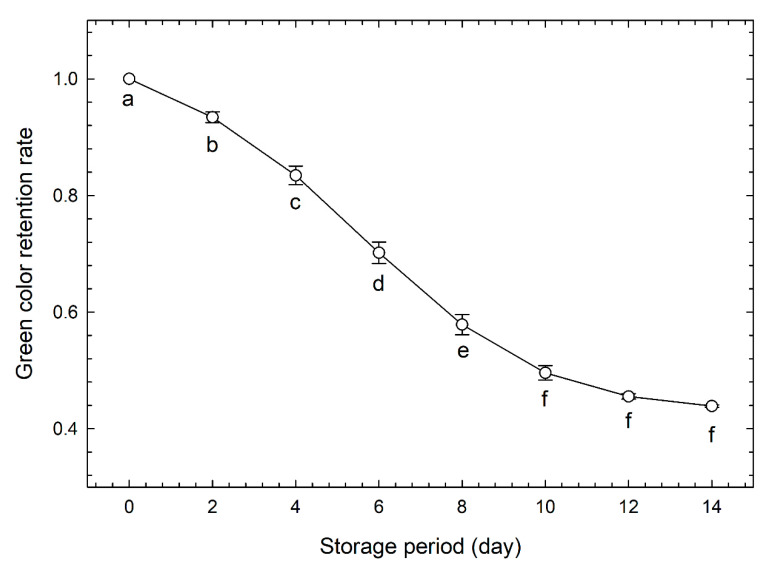
Changes in green color (−a*/b*) retention rates of broccoli heads at stored at 10 °C and 80% relative humidity over time. Values are the means ± SE of three observations from three biological samples (test data set). Symbols followed by the same letter within the same figure indicate no significant difference (*p* < 0.05, Tukey’s honest difference test).

Table 2 shows the results of the classification of the test data set by storage period length using the model created by PLS-DA of the calibration data set. In the classification (Table 2A), with green color retention and mass retention rates as the input variables, there were eight misclassifications, and the correct answer rate was 67%. Usually, the freshness of broccoli is evaluated by measuring the greenness of the flower buds [25,30,31]. Therefore, Figure 5 shows the changes over time in the green color retention rates of the test data set. Similar to the calibration data set, the green color retention rate decreased significantly every 2 days between day 0 and day 10; however, there was no significant difference in the values between day 10 and day 14. According to the results shown in Table 2A, a total of six misclassifications occurred on day 6 and day 8, and no superiority was recognized when compared with the freshness evaluation based only on the green color retention rate (Figure 5). When green color retention and the area retention rate were used as the input variables, there were 14 misclassifications, which was higher than when the mass retention rate was used as the input, and the accuracy rate dropped to 42%. The results of Table 2A,B were compared for the same storage period lengths. In the storage period length of 2 d, only (B) had two misclassifications. In the storage period length of 4 days, only (B) had three misclassifications. Day 6 had three misclassifications in (A) and (B). Day 8 had three misclassifications in (A). On 10 days, there were two misclassifications in (B), and there were two misclassifications in (A) and four misclassifications in (B) during a period of 12 days–14 days. Misclassification occurred at all time points except for day 0 and day 8. The above results demonstrate that it is difficult to obtain an effective freshness evaluation value by PLS-DA.

**Figure 6:**
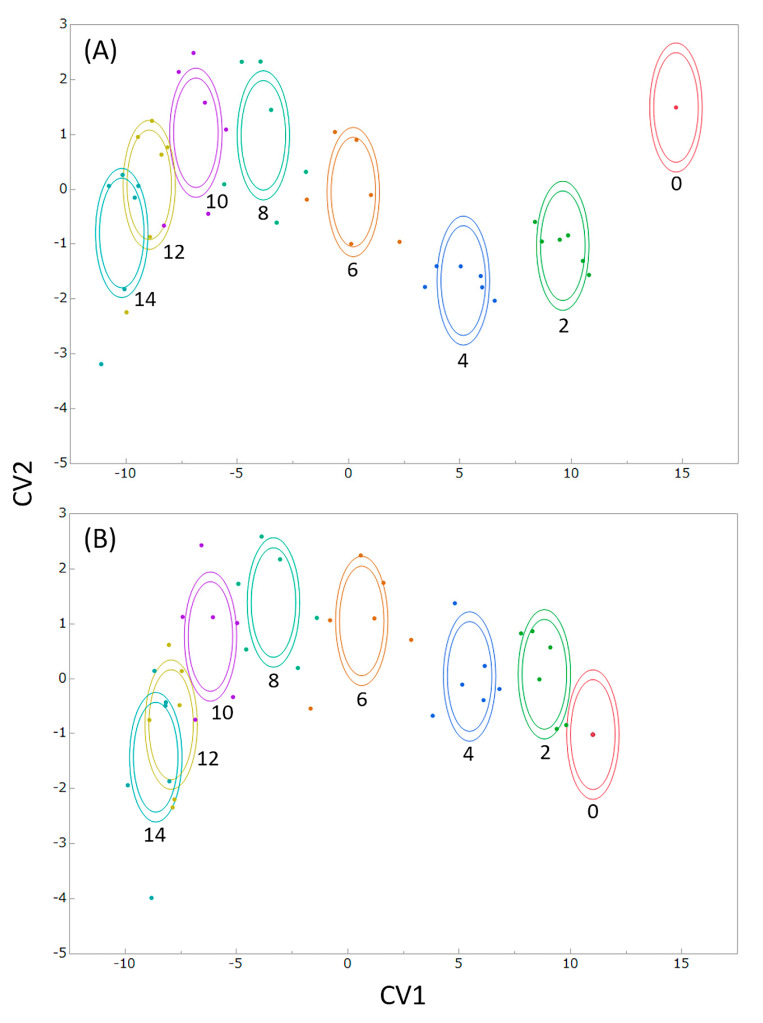
Discriminant analysis evaluation of the freshness of broccoli heads (calibration data set) stored at 10 °C and 80% relative humidity. (**A**) covariates: green color and mass retention rate, (**B**) covariates: green color and area retention rate, CV denotes the canonical variable, numbers in the figure denote the duration of storage in days, crosses denote the mean CV values of the same duration of storage, concentric circles denote the normal 50% contour lines (outer) and 95% confidence region for group mean (inner); the difference of colors of the plots and circles in this figure denotes the difference of storage period duration.

Figure 6A shows the results from LDA using the calibration data set of classification by storage period length (covariates: green color retention and mass retention rate). Although there were two freshness evaluation items, they were integrated into the canonical variable (CV) 1 and were effective for classification by storage period length. Figure 6B shows the results from LDA classified by storage period length (covariates: green color retention and area retention rate). Both covariates were integrated into CV1 and were effective in categorizing by the duration of the storage periods.

Table 3 shows the results of the classification of the test data set by storage period length using the model created by LDA of the calibration data set. There was one misclassification and the correct answer rate was 96% when the classification was done using green color retention rate and mass retention rate as covariates (Table 3A). As shown in Figure 5, the change in green color retention rate could not be detected in the period from 12 days to 14 days, however, the freshness evaluation value combined with the mass retention could generate a classification up to day 12. This suggests that it is possible to classify freshness with a higher degree of accuracy than when using only the green color retention rate (Figure 5). That is, it has become clear that a more accurate freshness evaluation is possible when the change in mass is also evaluated.

When classification was done using green color retention and area retention rate as covariates, there were four misclassifications, and the correct answer rate was 83%, which was lower than when mass retention was selected as a covariate (Table 3B). Misclassification occurred when the storage duration was between 10 days and 12 days, similar to the results from the freshness evaluation using green color retention alone (Figure 5). As shown in Figure 3, since the area retention rate is highly correlated with mass retention rate, it is possible to create a freshness evaluation index that integrates color and mass only by image capturing when these factors are combined with the green color retention rate. However, because the classification accuracy by storage period duration was similar to the evaluation conducted using only green color retention rate, the conventional method [25,30,31] was used. In contrast, when the mass retention rate determined by weighing was added, the storage period durations could be classified with higher accuracy even when yellowing had progressed, as compared with a freshness evaluation using only green color. Since freshness is closely related to the storage period duration, the freshness evaluation index is considered to be a suitable prediction method.

## 4. Discussion

Not all broccoli heads harvested on the same day will lose freshness at the same velocity, and it is, therefore, possible that the duration of storage can be incorrectly classified. However, the prediction accuracy of LDA (input color and mass loss) was the highest, with the prediction results similar to the actual storage durations, as indicated by the results from the same samples and same data set (Table 2 and Table 3). The classification based on LDA (input color and mass loss) was the best (Figure 4 and Figure 5).

As shown in Table 2A and Table 3, the misclassification rate was higher for samples with longer durations of storage. Although the fresh samples were classified clearly, it was difficult to classify the samples that had been stored for longer periods (Figure 4A and Figure 6). The method proposed in this study can evaluate freshness more effectively when the samples are fresher.

Since broccoli flower buds remarkably turn yellow with deterioration, some studies have been reported that objectively evaluate the freshness by numerically measuring the external color. One is a method using a colorimeter. Kasim et al. [37] treated broccoli heads with 1-methylcyclopropene, sealed them with polyvinyl chloride films, and stored for 28 days at 5 °C and R.H. 95–98%. The decrease in hue angle due to the yellowing of broccoli flower buds was evaluated objectively. Ren et al. [38] confirmed that the broccoli heads were unwrapped or packed with high density polyethylene pouch and stored at 5 °C for 25 days or 10 °C for 10 days, resulting in a decrease in hue angle due to yellowing of the flower buds. Makhlouf et al. [30] evaluated yellowing of broccoli heads stored at 1 °C for 6 weeks by measuring a decrease in −*a*/*b*. However, no statistically significant differences were shown in the previous reports mentioned above. Wang et al. [25] showed that green color (−a*/b*) of cut broccoli stored at 25 °C for 50.5 h was significantly reduced under normoxia over time. In contrast, spatial distribution of yellowing in broccoli was observed [20]. However, the colorimeter can only evaluate the color of the part where the sensor is attached, and it remains questionable whether the freshness of broccoli can be properly evaluated based on the spatial color change. Makino and Kousaka [32] evaluated the spatial distribution of yellowing in broccoli by hyperspectral imaging (HSI). However, since the HSI stores the spectral reflectance/absorbance for each pixel, it is necessary to analyze a huge amount of data, which may increase the time required for evaluation. In contrast, in the case of CVS, only three RGB wavelengths are stored for each pixel, which enables quick data processing and freshness evaluation. Makino et al. [31] reported that −a*/b* values of flower buds of broccoli heads stored at 25 °C for 5 days decreased with deterioration, and CVS could quantify the degree of yellowing.

Weighing as well as image capturing is non-destructive measurement methods. Kasim et al. [37], Makhlouf et al. [30], Wang et al. [25], Makino and Kousaka [32] also reported that mass loss or mass retention rate decreased as broccoli freshness declined. Although color and mass are the main freshness indicators of broccoli that can be measured nondestructively, they have been used as independent freshness indicators in the previous reports, and there are no studies on freshness evaluation using integrated evaluation values. As a result of this study, it has been shown that a novel non-destructive freshness evaluation index that uses image capturing and weighing together can evaluate freshness with higher accuracy than the conventional method of only external color assessment. Using a color evaluation stand as a balance, color and mass can be measured simultaneously, which can help retailers to objectively and easily measure the freshness of broccoli. We can help reduce food loss by identifying products with low freshness at retail stores early so they can be re-directed for use in cooked foods. However, the method proposed in this study is an effective index for commodities in which the green color decreases as freshness declines, and it is difficult to apply to commodities that do not experience loss of green color (e.g., spinach, bok choy) (Figure S1).

## 5. Conclusions

It is known that the freshness of vegetables decreases with time after harvest. In particular, the progress of yellowing of green vegetables is observed as a serious phenomenon of deterioration in freshness. For this reason, many studies have been published in the past that evaluated the freshness of green vegetables by surface color. In contrast, it is also known that the mass decreases as the freshness of vegetables decreases. Therefore, in this study, we selected broccoli, which shows significant yellowing and mass loss, as a sample, and stored it for 14 days at 10 °C and 80% R.H. External color and mass were measured with a CVS and an electronic balance, respectively. The green color retention rate and mass retention rate decreased with time, indicating that both variables as freshness evaluation indices are closely related to the number of days of storage. Furthermore, a decrease in the number of pixels in the image corresponding to the broccoli flower bud (the area of the flower bud) was observed over time, and the retention of pixel count and the mass retention were related by a non-linear mathematical model. As a result of analyzing the data by ANOVA, the number of storage days significantly affected green retention rate, mass retention rate, and area retention rate (*p* < 0.05). Furthermore, in this study, we proposed a novel freshness evaluation index that integrates broccoli yellowing and mass loss. When the PLS-DA model was applied to create the index by inputting green color and mass retention rates, the correct answer rate for predicting storage days was 67%, which was inferior to the estimation result using only green color retention rate as a conventional method. When the modeling method was changed to LDA, the correct answer rate was improved to 96%, and it was possible to estimate with higher accuracy than the estimation result using only green color retention rate. The freshness of vegetables has been successfully evaluated by a single index created by integrating external color and mass loss. In this study, it was confirmed that the integrated index had higher estimation accuracy of freshness than the conventional method.

## Figures and Tables

**Table 1:** One-way analysis of variance of the effect of storage period length on the properties of broccoli heads stored for 14 days at 10 °C and 80% relative humidity.

Variable	Effect	Degree of Freedom	Sum of Square	*F*	*p*
Green color retention rate	Storage period	7	2.01	309	<0.001 ***
	Error	40	0.0371		
	Total	47	2.05		
Mass retention rate	Storage period	7	1.13	261	<0.001 ***
	Error	40	0.0248		
	Total	47	1.16		
Area retention rate	Storage period	7	0.223	60.6	<0.001 ***
	Error	40	0.0210		
	Total	47	0.244		

*** Significant at 99.9% level.

**Table 2:** Prediction results from partial least square discriminant analysis on the duration of storage of broccoli heads (test data set, three heads per day) stored at 10 °C and 80% relative humidity.

(A) Covariates: Green Color and Mass Retention Rate								
Ac(days)	Pr(days)							
	0	2	4	6	8	10	12	14
0	3	0	0	0	0	0	0	0
2	0	3	0	0	0	0	0	0
4	0	0	3	0	0	0	0	0
6	1	0	2	0	0	0	0	0
8	0	0	0	0	0	3	0	0
10	0	0	0	0	0	3	0	0
12	0	0	0	0	0	1	2	0
14	0	0	0	0	0	0	1	2
**(B) Covariates: Green Color and Area Retention Rate**								
Ac(days)	Pr(days)							
	0	2	4	6	8	10	12	14
0	3	0	0	0	0	0	0	0
2	2	1	0	0	0	0	0	0
4	2	0	0	1	0	0	0	0
6	0	0	0	0	3	0	0	0
8	0	0	0	0	3	0	0	0
10	0	0	0	0	2	1	0	0
12	0	0	0	0	0	1	0	2
14	0	0	0	0	0	1	0	2

Ac: actual class; Pr: predicted class; black cells indicate misclassification.

**Table 3:** Discriminant analysis prediction results from storage period duration of broccoli heads (test data set, three heads per day) stored at 10 °C and 80% relative humidity.

(A) Covariates: Green Color and Mass Retention Rate								
Ac(days)	Pr(days)							
	0	2	5	6	8	10	12	14
0	3	0	0	0	0	0	0	0
2	0	3	0	0	0	0	0	0
4	0	0	3	0	0	0	0	0
6	0	0	0	3	0	0	0	0
8	0	0	0	0	3	0	0	0
10	0	0	0	0	0	3	0	0
12	0	0	0	0	0	0	3	0
14	0	0	0	0	0	0	1	2
**(B) Covariates: Green Color and Area Retention Rate**								
Ac(days)	Pr(days)							
	0	2	4	6	8	10	12	14
0	3	0	0	0	0	0	0	0
2	0	3	0	0	0	0	0	0
4	0	0	3	0	0	0	0	0
6	0	0	0	3	0	0	0	0
8	0	0	0	0	3	0	0	0
10	0	0	0	0	1	2	0	0
12	0	0	0	0	0	1	1	1
14	0	0	0	0	0	0	1	2

Ac: actual class; Pr: predicted class; black cells indicate misclassification.

## Supplementary Materials

The following are available online at https://www.mdpi.com/2304-8158/9/9/1305/s1, Figure S1: Changes in green color retention (−*a**/*b**) rate of (A) spinach and (B) bok choy at 20 °C and 27.5% relative humidity over time.
