# Supplementary figures and images for: Digitization of Broccoli Freshness Integrating External Color and Mass Loss
Source: Foods. 2020 Sep 16;9(9):1305. doi: 10.3390/foods9091305 (PMC7554949; doi:10.3390/foods9091305)

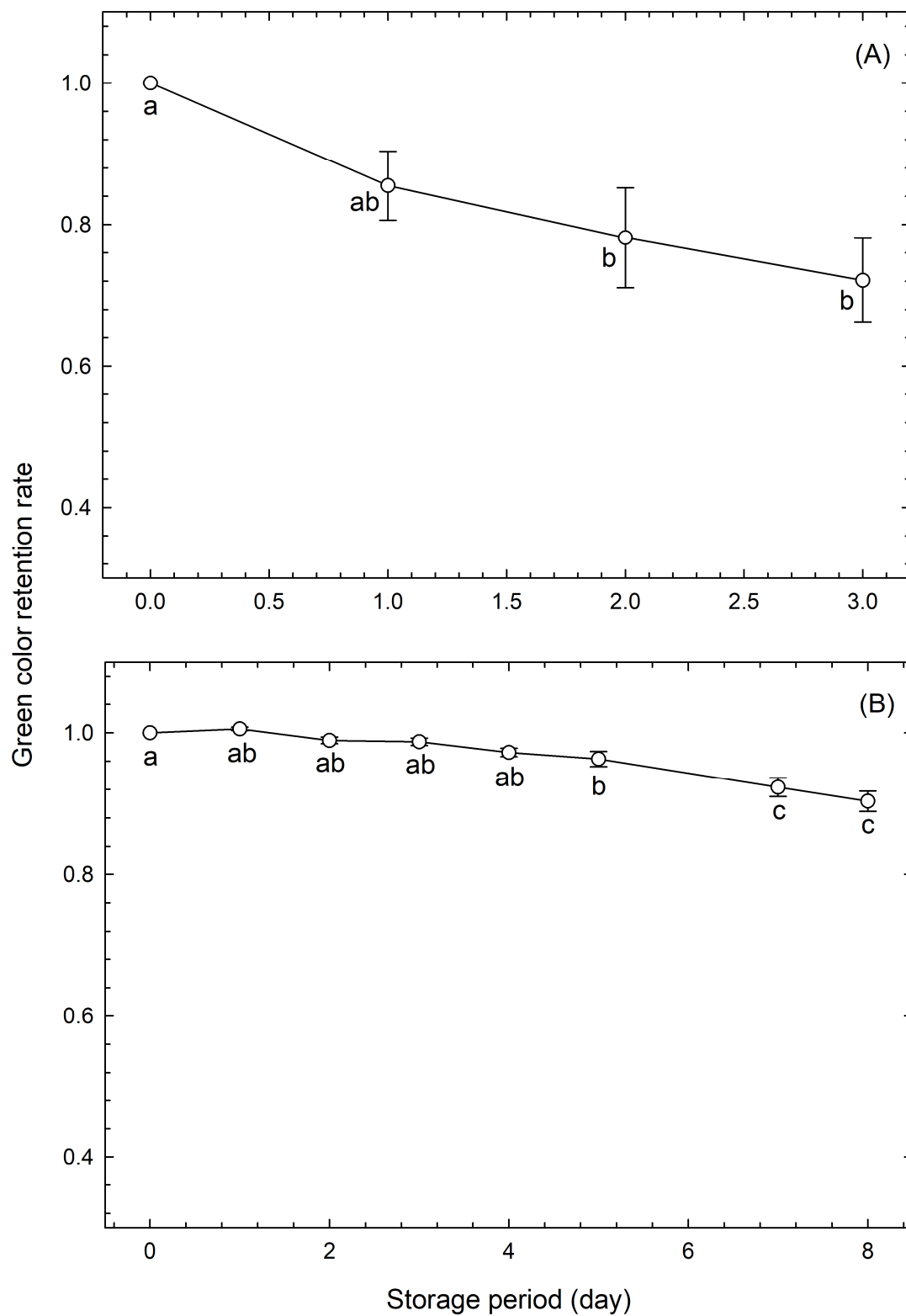

Supplement: Supplementary file 1 [file foods-09-01305-s001.pdf]
